# Physiologic Concentrations of HMGB1 Have No Impact on Cytokine-Mediated Eosinophil Survival or Chemotaxis in Response to Eotaxin-2 (CCL24)

**DOI:** 10.1371/journal.pone.0118887

**Published:** 2015-03-16

**Authors:** Kimberly D. Dyer, Helene F. Rosenberg

**Affiliations:** Inflammation Immunobiology Section, National Institute of Allergy and Infectious Diseases, National Institutes of Health, Bethesda, Maryland, United States of America; Universidade Federal do Rio de Janeiro, BRAZIL

## Abstract

HMGB1 is an alarmin that can stimulate the innate immune system alone or in a complex with other inflammatory mediators. Given the recent interest in HMGB1 with respect to the pathogenesis of eosinophil-associated disorders, including asthmatic inflammation and chronic rhinosinusitis, we have explored the role of this mediator and in promoting eosinophil activation. HMGB1 receptors RAGE and TLR4 but not TLR2 were detected on freshly isolated human eosinophils from healthy donors. Physiologic and relevant pathophysiologic levels of biologically-active HMGB1 had no effect on survival of human eosinophils alone or in combination with pro-survival cytokines IL-5, IL-3, or GM-CSF, and increasing concentrations of HMGB1 had no impact on surface expression of RAGE, TLR2 or TLR4. Similarly, HMGB1 did not elicit chemotaxis of human eosinophils alone and had no effect in combination with the eosinophil chemotactic agent, eotaxin-2 (CCL24). However, surface expression of TLR2 and TLR4 increased in response to cell stress, notably on eosinophils that remain viable after 48 hours without IL-5. As such, HMGB1 signaling on eosinophils may be substantially more detailed, and may involve complex immunostimulatory pathways other than or in addition to those evaluated here.

## Introduction

High mobility group box 1 protein (HMGB1) is an evolutionarily ancient protein that was originally characterized as a chromatin stabilizing nuclear DNA-binding protein. Wang and colleagues [1] were the first to identify an extracellular role for HMGB1, specifically its participation in cellular activation and pro-inflammatory responses (reviewed in [2–10]). Containing 215 amino acids comprising three distinct protein domains, HMGB1 is expressed ubiquitously, is released from dead and dying cells, and serves as an alarmin or damage-associated molecular pattern (DAMP) molecule, stimulating the innate immune system by itself or via immunostimulatory complexes with endotoxin, nucleic acids, or proinflammatory cytokines and chemokines [3,11]. Additionally, activated immune cells (macrophages, monocytes, dendritic cells and natural killer cells) and endothelial cells secrete HMGB1 in response to pro-inflammatory stimuli [2].

HMGB1 signals through multiple surface receptors; TLR2, TLR4, and RAGE, receptor for advanced glycation end product, are the best characterized [2,12] but HMGB1 can also signal through human CD24 / Siglec-10 [13]. A series of recent studies has revealed a role for HMGB1 in sensing and responding to exogenous and endogenous nucleic acids (double-stranded RNA, single-stranded RNA, CpG-containing oligodeoxynucleotides) and amplifying the responses of these ligands to pattern recognition receptors TLR3, TLR7, and TLR9 (reviewed in [9]). Interestingly, while HMGB1 gene-deleted mice die in infancy [14], mice with conditional ablation of HMGB1 in myeloid cells develop normally, although they are more sensitive to endotoxin shock compared with control mice [15].

There is substantial interest in HMGB1 signaling and inflammation associated acute and chronic disease, notably in diseases associated with eosinophilic inflammation [7,16]. Elevated levels of HMGB1 have been detected in sputum, plasma and nasal lavage of eosinophilic asthmatics as compared to normal controls, with levels of HMGB1 correlating with both sputum levels of IL-5, IL-13, and eosinophil counts [17–19]. Additionally, there is a negative correlation between HMGB1 levels and pulmonary function [20]. Similarly, HMGB1 has been implicated in the pathogenesis of chronic rhinosinusitis, an asthma co-morbidity characterized by eosinophils in nasal polyps and in mucous drainage [21]. Expression of HMGB1 was detected in paranasal sinus mucosae of individuals with this condition [22,23], with levels correlating directly with those of serum IL-5 and blood eosinophil counts [24].

HMGB1 expression is ubiquitous and serum levels in normal individuals are on the order of <5–30 ng/ml but can rise 3-fold or more under conditions associated with eosinophil activation and recruitment (**Table 1**). Lotfi *et al*. [25] demonstrated that human eosinophils are mobilized and activated in response to supraphysiologic concentrations of HMGB1 (10^4^ ng/ml) and proposed a role for this DAMP in the induction of eosinophilic inflammation. At these supraphysiologic concentrations, which are similar to those described by Ueno *et al*. [26] in lung epithelial lining cells responding to acute lung injury in response to sepsis, HMGB1 promoted survival, chemotaxis and degranulation of eosinophils. We were interested in expanding the findings of Lotfi *et al*. [25] and in exploring the effects of HMGB1 on human eosinophils at concentrations similar to those identified in acute and chronic states directly associated with eosinophilic disease. Additionally, we wanted to explore the role of HMGB1 in combination with eosinophil active cytokines as it is known that HMGB1 can form immunostimulatory complexes with other soluble mediators. Here, we examine the expression of HMGB1 receptors on human eosinophils isolated from peripheral blood and explore the impact of HMGB1 in promoting eosinophil chemotaxis and survival.

**Table 1:** HMGB1 concentrations associated with inflammatory and eosinophil-related diseases.

Disease	Reference	Body fluid	Conditions evaluated	[HMGB1] ng/mL
Vasculitis	[18]	Serum	Controls	30 ± 16
			Urticarial vasculitis	51 ± 32
			Allergic vasculitis	53 ± 37
			Henoch-Schonlein purpura	57 ± 41
	[27]	Serum	Controls	< 5
			Wegener's granulomatosis	12 ± 9
Dermatitis	[28]	Serum	Controls	28 ± 18
			Psoriasis	78 ± 66
			Atopic dermatitis	35 ± 20
Allergy/Asthma	[29]	Nasal lavage	Controls	9 ± 4
			Allergic rhinitis	97 ± 19
	[20]	Plasma	Controls	3.7
			Eosinophilic asthma	4.7
	[20]	Sputum	Controls	0.4
			Eosinophilic asthma	3.8
Autoimmune	[30]	Serum	Controls	13 ± 10
			Lupus nephritis	108 ± 48
	[31]	Serum	Controls	18
			Rheumatoid arthritis	71

## Materials and Methods

### Isolation of eosinophils from normal donors

One hundred milliliter (100 mL) samples of peripheral blood from normal donors were obtained on the Laboratory of Allergic Diseases normal donor protocol (NIAID Protocol Number: NIH 09-I-0049). This protocol is designed to assure adequate and complete informed consent, counseling, and protection of the study subjects according the NIAID Institutional Review Board, Office of Human Subjects Research (OHSR), Office for Human Research Protections (OHRP) and other applicable Federal regulatory standards. As part of this protocol, written consent is obtained from each normal volunteer allowing their sample to be used in this study. Eosinophils were isolated from the blood according to the protocol of Percopo *et al*. [32]. Essentially granulocytes were separated on a 67% Percoll gradient (GE #17-0891-02) and eosinophils were isolated by negative selection using the Miltenyi eosinophil isolation kit as per manufacturer’s directions. The eosinophils were either used immediately or cultured for 48 hours in the presence or absence of the anti-apoptotic cytokine, IL-5 (100 pg/mL) with or without HMGB1 as indicated. The monocytic cell fraction from the Percoll gradient was collected and used to determine the titer of the anti-human TLR2 (CD282) and anti-human TLR4 (CD284) antibodies. Additionally, the granulocytes, prior to isolation of eosinophils, were fixed in 4% paraformaldehyde (EMS, Hatfield, PA) and used to determine the titer of the anti-human RAGE antibody.

### Flow cytometry

Non-specific antibody binding was blocked by incubating human eosinophils with FcR blocking reagent (Miltenyi). Antibody directed against the indicated HMGB1 receptor—RAGE, TLR2 or TLR4 is shown in **Table 2**. After incubation with antibodies for 30 minutes at 4°C, the cells were washed with 3 ml of 0.1% BSA/PBS and then fixed in 4% paraformaldehyde. The samples were stored at 4°C in the dark until analyzed. A minimum of 100,000 events was collected on an LSRII flow cytometer (BD Biosciences) and data was analyzed in FlowJo (Tree Star, Inc). Gating was performed by comparison to isotype control for each antibody. Isotype controls (fluorescence minus one) were subtracted from each data point.

**Table 2:** Antibodies used to examine surface expression of HMGB1 receptors on human eosinophils.

Antigen	CloneVendor	Concentration(10^6^ cells)	Notes
TLR2/CD282	TL2.1 Ebioscience	0.5 μg / 100 μL	Titered on monocytes
TLR4/CD284	HTA125 Ebioscience	0.5 μg / 100 μL	Titered on monocytes
RAGE	176907 R&D	0.5 μg / 100 μL	Used on 4% paraformaldehyde fixed cells; Secondary rat anti-mouse IgG1—APC

### HMGB1 activity assay

This assay was modified from that described by Gero *et al*. [33] RAW264.7 (ATCC, TIB71) were plated at 100,000 cells per well in a 96-well plate in 200 μL of media containing RPMI1640 (Life Technologies), 10% FBS (Lonza), 100 IU/mL penicillin, and 10 μg/mL streptomycin (Life Technologies) and grown overnight at 37°C in a cell culture incubator. The culture media was removed and 100 μL Optimem (Life Technologies) containing 0–100 ng/mL HMGB1 (R&D Systems) was added. Lipopolysaccharide (LPS; Sigma) was used at 50 ng/mL as a positive control. After 7 hours, the plate was centrifuged at 300 x g for 5 minutes at room temperature to remove the cells and the cell free supernatant was stored at −80°C. The samples were diluted 1:10 in Optimem prior to determining TNFα concentrations by ELISA (R&D) according the manufacturer’s recommendations. We determined that recombinant HMGB1 used in our studies was biologically active, with 100 ng/mL eliciting 177 pg/mL TNF-alpha, ∼3-fold over background levels at t = 7 hrs.

### Viability assay

Eosinophils were suspended in media with the indicated cytokines (HMBG1, IL-3, IL-5 and GM-CSF; R&D) and were incubated for in a 37°C in a humidified CO_2_ incubator for 48 hours. The cells were washed one time in 3 mL PBS (Life Technologies) and stained with Violet live-dead (Life Technologies) for 30 minutes in the dark. The samples were washed with 3 mL 0.1% BSA/PBS and fixed with 200 μL 4% paraformaldehyde at 4°C. Prior to running the samples on a LSRII flow cytometer, they were washed once to remove the paraformaldehyde and suspended in 100 μl 0.1% BSA/PBS. Total cell number per sample and percent viability was obtained.

### Chemotaxis Assay

The chemotaxis assay was performed as previously described [34]. Briefly, eosinophils were suspended at 10^6^/mL in chemotaxis buffer and 100 μL was placed in the top well of a 5-μm 96-well Transwell plate (Corning). The 100 μL of the chemoattractant, diluted in chemotaxis buffer, was placed in the bottom well. The plate was incubated for 3 hours at 37°C in a humidified CO_2_ incubator. Cells that migrate to the lower well through the membrane are collected and enumerated by collecting events on LSR II at high flow rate for 60 seconds. Data is reported as cell number or chemotactic index (CI = cells migrating in response to chemoattractant / cells migrating in response to vehicle). Recombinant human eotaxin (rhE2, R&D) was used as a positive control for eosinophil chemotaxis.

## Results and Discussion

### Expression of HMGB1 receptors on eosinophils

**Fig 1:**
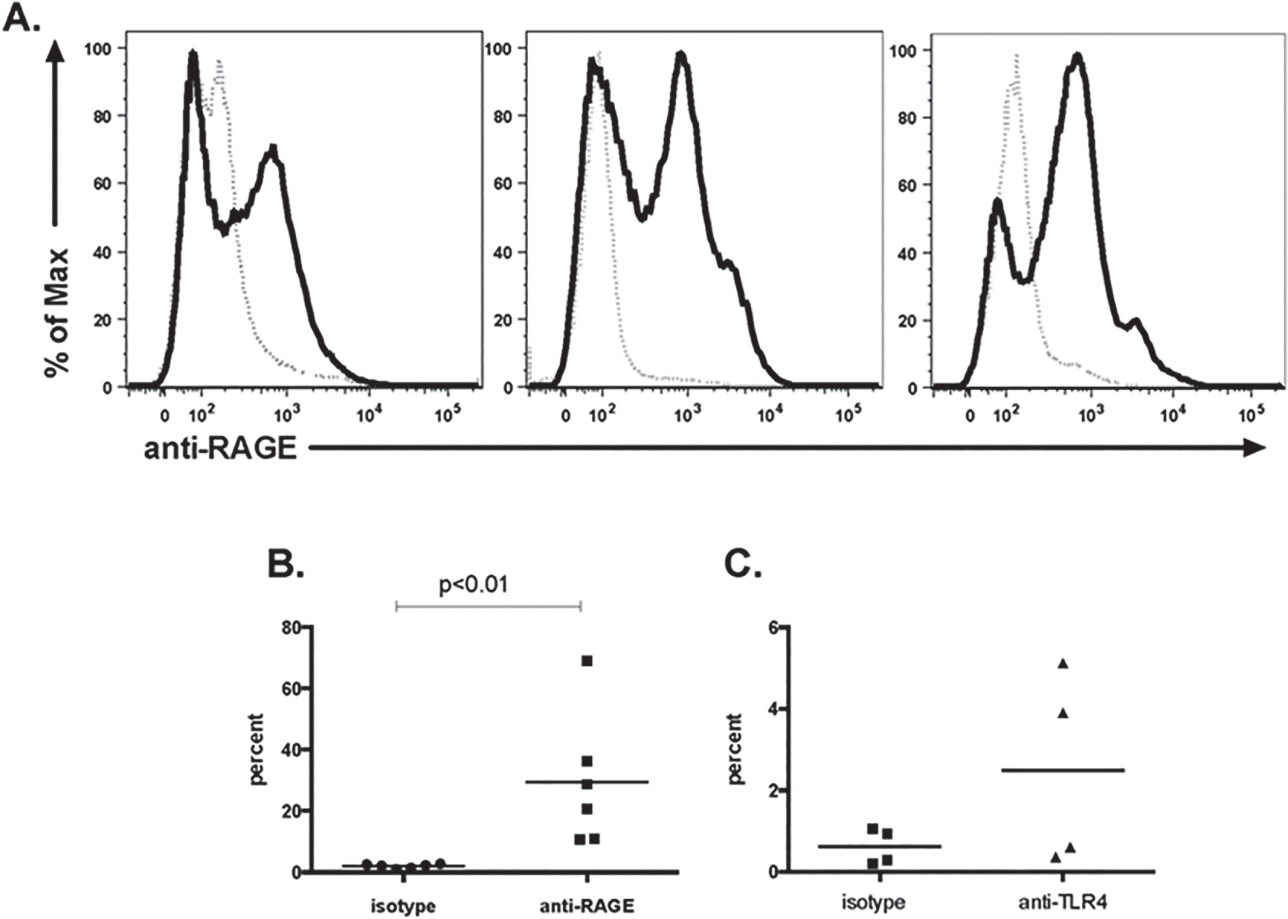
Expression of HMGB1 receptors on eosinophils. Human eosinophils were isolated from peripheral blood of normal donors. Surface expression of RAGE and TLR4 was evaluated by flow cytometry. **(A)** RAGE expression (heavy solid line) in comparison to isotype control (dotted line) in three individual donors. **(B)** RAGE expression in from 6 normal donors shown in comparison to isotype control. **(C)** TLR4 expression in from 4 normal donors shown in comparison to isotype control.

Human eosinophils were isolated from the peripheral blood of normal donors and surface expression of RAGE, TLR2 and TLR4 was examined. RAGE expression was variable among normal donors and on average 30% of the eosinophils isolated from normal human donors express RAGE (range 11–69%, n = 6 donors, **Fig. 1A** and **1B**). The expression of pattern recognition receptors on eosinophils has recently been reviewed [4]. Our results are consistent with those of others who have shown documented expression of RAGE on human eosinophils [25,35]; however, this is the first study documenting the heterogeneous nature of RAGE expression on eosinophils isolated from multiple normal donors.

TLR4 was detected on a small population (0.3–5%) of eosinophils from normal donors **(Fig. 1C).** Our findings are consistent with those of Wong *et al*. [36] who detected TLR4 by both Western blot and flow cytometry. In contrast, we did not detect TLR2 on freshly isolated normal human eosinophils. Our findings are consistent with those of Nagase *et al*. [37] and Sabroe *et al*. [38] who were unable to detect surface expression of TLR2 by flow cytometry. Interestingly Wong *et al*. [36] reported detection of TLR2 on and in human eosinophils with intracellular expression being higher then surface expression. Likewise Wong *et al*. [36] reported that peptidoglycan (PGN), a TLR2 ligand, signaled through this receptor; in contrast, even though TLR4 was present, its ligand LPS did not elicit a response. Similarly, Sabroe *et al*. [26] failed to elicit a response from eosinophils with LPS stimulation.

**Fig 2:**
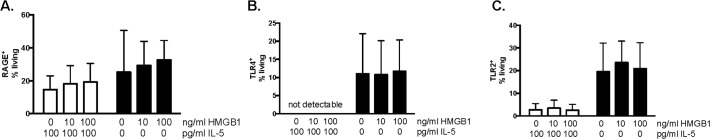
HMGB1 has no impact on cell surface expression of RAGE, TLR2 or TLR4. Eosinophils isolated from peripheral blood of normal donors incubated in the presence (open bars) or absence (closed bars) of IL-5 with 0, 10 or 100 ng/ml HMGB1for 48 hours and then evaluated for the expression of **(A)** RAGE, **(B)** TLR4, and **(C)** TLR2. Data represent mean ± SEM of percent population of live eosinophils that are positive for indicated receptor minus staining with isotype control; n = 3 independent donors.

Eosinophils were isolated from peripheral blood of normal donors and cultured for 48 hrs either in the presence or absence of IL-5; living cells (87.9 ± 2.0% of total in response to 100 pg/mL IL-5 culture and 42.1 ± 5.9% in the absence of IL-5) were evaluated for the expression of RAGE, TLR4, and TLR2. At the same time, both cultures were treated with increasing concentrations of HMGB1 (from 0 to 100 ng/mL). As shown, HMGB1 had no independent effect on the cell surface expression of RAGE, TLR4 or TLR2. In contrast, IL-5 had a clear and differential impact on receptor expression. While IL-5 had little to no effect on the surface expression of RAGE on live eosinophils (**Fig. 2A**), this was not the case for surface expression of TLR4 (**Fig. 2B**) or TLR2 (**Fig. 2C**). Detection was minimal (TLR2) to non-existent (TLR4) after 48 hrs in culture with 100 pg / mL IL-5. In contrast, immunoreactive TLR2 and TLR4 was detected on ∼20% and ∼10% of the live cells, respectively, after 48 hrs in cultures devoid of IL-5. This finding is intriguing and requires further study.

### Recombinant human HMGB1 does not enhance viability or chemotaxis of isolated human eosinophils

As noted in the Introduction, very high concentrations (10^4^ ng/mL), were utilized in an earlier publication documenting HMGB1-mediated eosinophil activation [25]. This concentration exceeds physiologic serum levels (typically measured at 0–30 ng/mL), and likewise exceeds levels measured in serum, plasma and tissue fluids of individuals with eosinophil-associated disorders (**Table 1**).

**Fig 3:**
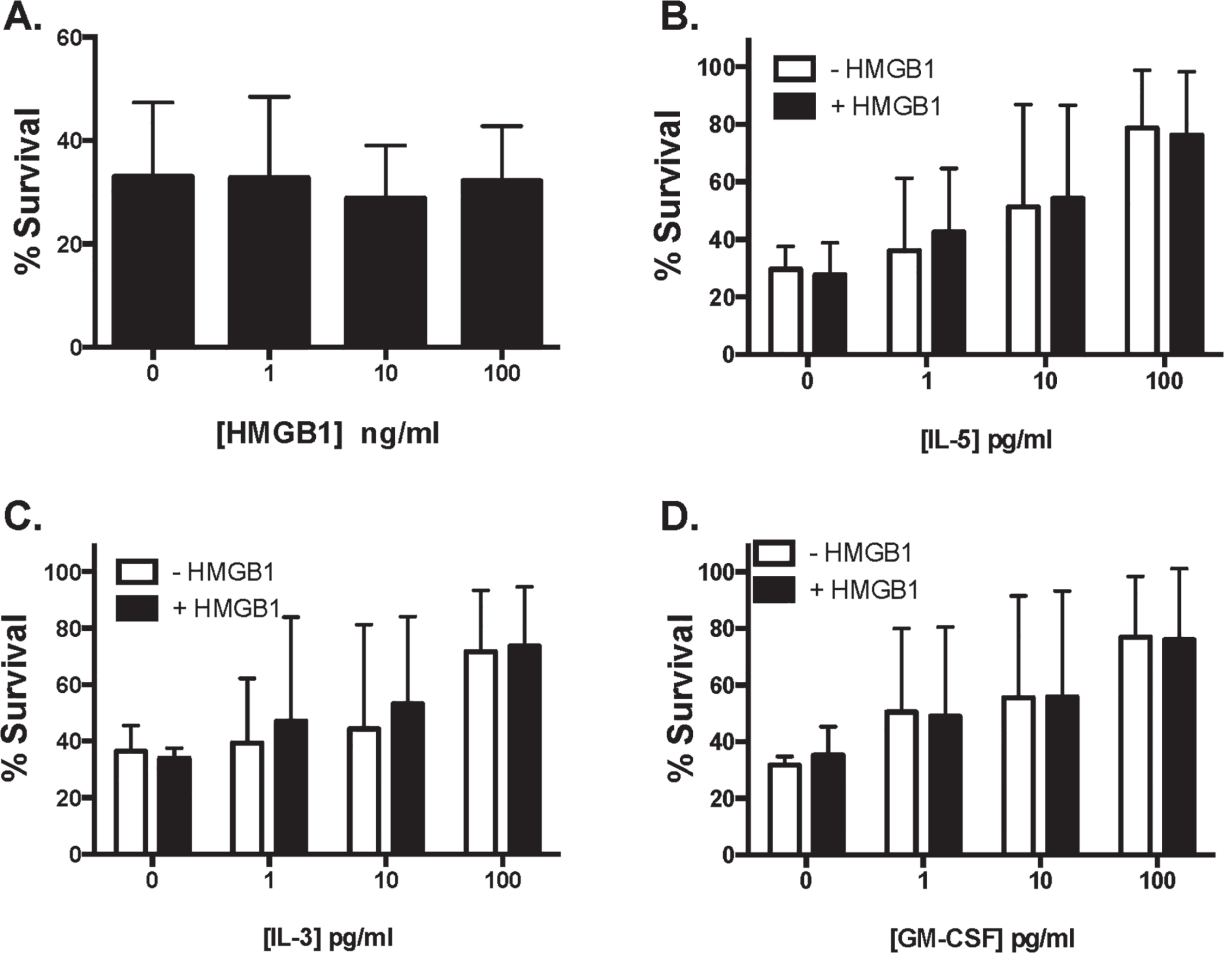
HMGB1 has no impact on eosinophil survival *ex vivo*. Eosinophils isolated from peripheral blood of normal donors incubated with **(A)** HMGB1 alone or 10 ng/mL HMGB1 in combination with increasing concentrations of pro-survival cytokines **(B)** IL-5, **(C)** IL-13 or **(D)** GM-CSF for 48 hours and then evaluated with Violet live-dead for the assessment of viability.

When evaluated alone, in the absence pro-survival cytokines (ie, under conditions of pro-apoptotic stress [39]), HMGB1 had no impact on survival of human eosinophils (**Fig. 3A**). Similarly, HMGB1 at 10 ng/mL had no impact on eosinophil survival promoted by IL-5, IL-3, or GM-CSF **(Fig. 3B-D).**

**Fig 4:**
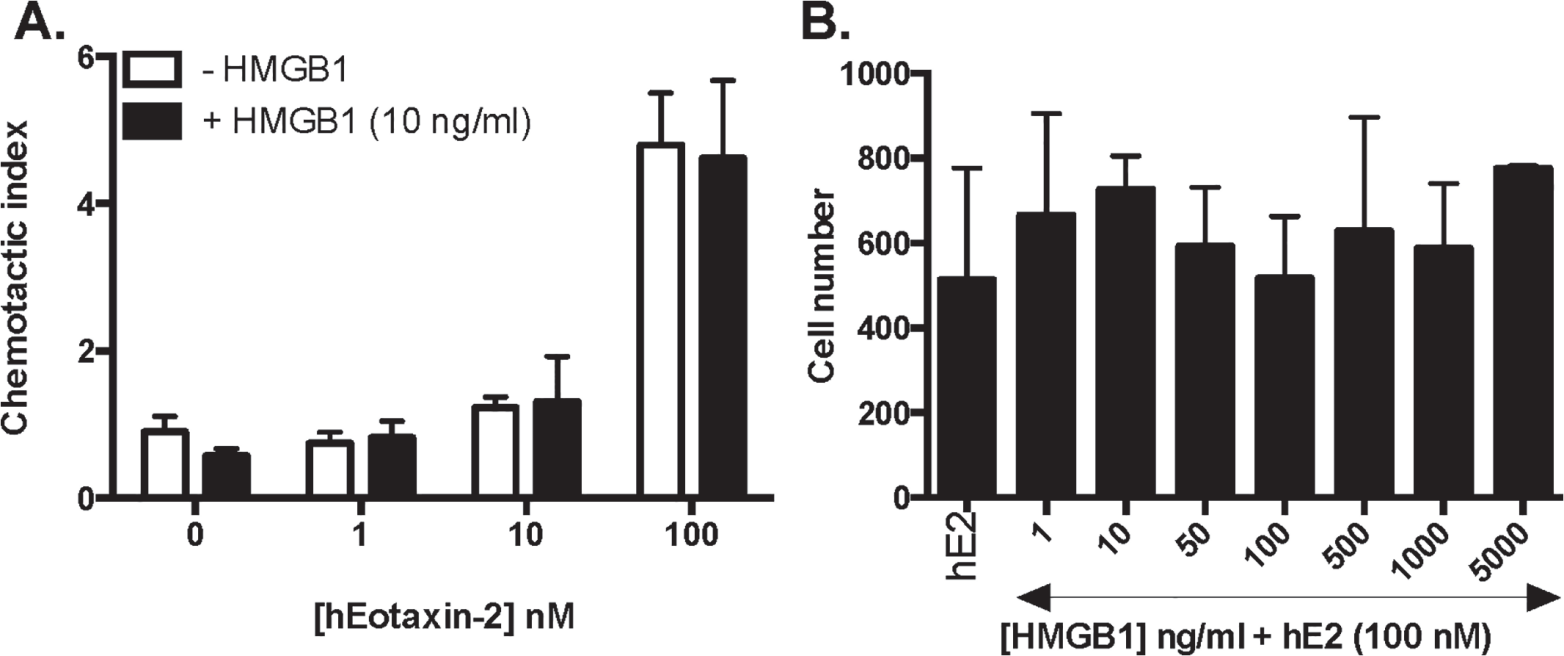
HMGB1 has no impact on eotaxin-2-mediated chemotaxis. Chemotaxis of peripheral blood eosinophils from normal donors evaluated in response to **(A)** increasing concentrations of human eotaxin-2 in the presence or absence of 10 ng/mL HMGB1 or **(B)** 100 nM human eotaxin-2 (hE2) in the presence of increasing concentrations of HMGB1.

Likewise, as shown in **Fig. 4A**, HMGB1 at 10 ng/ml elicited no chemotaxis of human eosinophils and had no impact on chemotaxis elicited by the eosinophil chemotactic agent, eotaxin-2 (CCL24). Eosinophils incubated for 30 minutes with HMGB1 prior to introduction of an eotaxin-2 gradient migrated to the same extent as did the eosinophils pre-incubated with buffer alone. Furthermore, varying concentrations of HMGB1, including supraphysiologic concentrations (1–5000 ng/ml) had no impact on chemotaxis elicited by 100 nM eotaxin **(Fig. 4B)**. Interestingly, this is in direct contrast to the inhibitory effect of HMGB1 observed when measuring neutrophil chemotaxis mediated via RAGE both at baseline at moderate concentrations (50–100 ng/mL) and in response to these concentrations in the presence of gradients of neutrophil-specific chemoattractants fMLP- and IL-8 [40].

## Summary

At physiologic and pathophysiologic concentrations related to eosinophil-associated disease, recombinant HMGB1 has no impact on survival of freshly isolated human eosinophils, or on the expression of its receptors, RAGE, TLR2 or TLR4, either alone or in conjunction with IL-3, IL-5, or GM-CSF, in experiments performed *ex vivo*, although a greater fraction of viable cells express TLR2 and TLR4 in cultures undergoing pro-apoptotic stress. Additionally, HMGB1 has no apparent impact on eotaxin 2-mediated chemotaxis, in direct contrast to what has been reported for HMGB1 and human neutrophils [40]. HMGB1 also did not have an impact on cytokine-mediated survival over a wide range of concentrations when used in combination with known eosinophil-active cytokines. From our findings, we must consider the possibility that HMGB1 may be generating complexes *in vivo* with other cytokines or with nucleic acid ligands that modulate chemotaxis and prolong survival and/or that may have an impact on eosinophil biology via outcomes other than those explored here.
